# Kinesiophobia and associated factors in postmenopausal osteoporosis: a controlled study

**DOI:** 10.1590/1806-9282.20241042

**Published:** 2024-12-02

**Authors:** Büşra Şirin Ahısha

**Affiliations:** 1 Beylikdüzü State Hospital – İstanbul, Turkey.

**Keywords:** Osteoporosis, Kinesiophobia, Quality of life, Depression, Anxiety

## Abstract

**OBJECTIVE:**

This study aimed to evaluate kinesiophobia levels in patients with osteoporosis compared to healthy controls and investigate the associations with pain, depression, anxiety, fear of falling, and quality of life.

**METHODS:**

The study involved 60 postmenopausal osteoporosis patients and 60 healthy controls aged 50 years and above. Kinesiophobia was assessed using the Tampa Scale for Kinesiophobia, while quality of life, psychological symptoms, and fear of falling were evaluated using the Quality of Life Questionnaire of the European Foundation for Osteoporosis, Hospital Anxiety and Depression Scale, and Tinetti Falls Efficacy Scale.

**RESULTS:**

Kinesiophobia levels were significantly higher in the osteoporosis group compared to controls (p<0.05). Positive correlations were observed between kinesiophobia and Falls Efficacy Scale (r=0.278, p=0.002), as well as with Quality of Life Questionnaire of the European Foundation for Osteoporosis physical function scores (r=0.185, p=0.043). No significant relationship was found between kinesiophobia and depression or anxiety scores.

**CONCLUSION:**

Kinesiophobia is notably higher in osteoporosis patients compared to healthy individuals, correlating with an increased fear of falling and reduced physical function. Early identification and management of kinesiophobia are essential to prevent reduced physical activity and associated risks, such as decreased bone mineral density and higher fracture risk.

## INTRODUCTION

Osteoporosis is a systemic metabolic disorder that leads to decreased bone mineral density and a higher risk of fractures, especially in older adults and women. Fragility fractures often occur in the vertebrae, hip, and distal radius, resulting in increased morbidity, mortality, and a significant economic burden on healthcare systems^1^. Low physical activity contributes to decreased bone mineral density and higher fracture risk from falls. Physical activity helps maintain bone mass and stimulates bone development, highlighting its importance in osteoporosis treatment and prevention^2^.

In patients diagnosed with osteoporosis, irrational fear of movement and physical activity, known as kinesiophobia, and avoidance behaviors can develop due to the thought that their bones are weak and prone to fractures. This situation leads to restrictions in physical activity and, in a vicious cycle, results in decreased bone density and consequently an increased risk of fractures^3^. Kinesiophobia is a condition encountered in many groups within the field of rehabilitation, including those with fibromyalgia^4^, chronic low back pain^5^, older individuals^6^, and systemic lupus erythematosus^7^. Kinesiophobia limits daily activities, impairs pain management, hampers rehabilitation, and delays return to sports and work. This leads to reduced physical performance, cardiac issues from insufficient activity, muscle loss, emotional problems, and worsened quality of life^6^. In the literature, fear of falling has been more extensively studied in patients with osteoporosis, with a limited number of studies evaluating kinesiophobia. The aim of this study is to fill this gap in the literature by investigating the presence of kinesiophobia in postmenopausal osteoporosis patients and comparing the level of kinesiophobia with healthy controls. Additionally, the relationship between the level of kinesiophobia and symptoms of anxiety and depression, fear of falling, and quality of life has been evaluated.

## METHODS

Our clinical cross-sectional study included 60 postmenopausal women with osteoporosis and 60 healthy postmenopausal women, recruited from Beylikdüzü State Hospital’s Physical Therapy and Rehabilitation clinics between January 21, 2024, and February 21, 2024. The study was approved by the Istanbul Physical Therapy and Rehabilitation Training and Research Hospital Ethics Committee (protocol number 2023-03) and obtained informed consent from all participants. The study adhered to the Helsinki Declaration principles. Bone mineral density was measured using dual-energy X-ray absorptiometry, classifying osteoporosis as lumbar L1–L4 and femoral neck T-scores of ≤-2.5, osteopenia as -1 to -2.5, and normal as >-1^8^.

Inclusion criteria were a diagnosis of postmenopausal osteoporosis for at least six months, exclusion of secondary causes, and age 50 years or older. Exclusion criteria included other conditions causing kinesiophobia, cognitive or communication issues, acute pain, history of falls and fractures, and conditions affecting balance or function.

At the beginning of the study, sociodemographic data, comorbidities, smoking habits, and sports activities were recorded. To assess the presence and level of kinesiophobia, the Turkish version of the Tampa Scale for Kinesiophobia (TSK)^9^ was used. Quality of life was assessed using the Turkish version of the Quality of Life Questionnaire of the European Foundation for Osteoporosis (QUALEFFO-41)^10^. Depression and anxiety were measured with the Hospital Anxiety and Depression Scale (HADS)^11^, and fear of falling was evaluated using the Tinetti Falls Efficacy Scale (TFES)^12^.

### The Tampa Scale for Kinesiophobia

The Tampa Scale for Kinesiophobia (TSK) was developed by Miller et al.^13^. The Turkish version of this scale, which was validated and found reliable in 2011, consists of 17 items that inquire about thoughts related to injury and physical activity. Each item is scored from 1 (strongly disagree) to 4 (strongly agree). Items 4, 8, 12, and 16 are reverse-scored. Total scores range from 17 to 68, with higher scores indicating higher levels of kinesiophobia^9^.

### Quality of Life Questionnaire of the European Foundation for Osteoporosis

The Turkish version of this scale, translated by Koçyigit et al.^10^, consists of four categories: pain, physical function, general health, and mental health. Separate scores for each category and a total score are calculated. The scores range from 0 to 100, with higher scores indicating poorer quality of life in that domain^10^.

### Hospital Anxiety and Depression Scale

The Turkish version of the HADS was validated and found reliable by Aydemir et al.^11^. This scale consists of a total of 14 items, with 7 items assessing anxiety symptoms and 7 items assessing depression symptoms. Each item is scored between 0 and 3. Higher scores indicate higher levels of anxiety and depression. The cutoff value is defined as 7 for the depression subscale and 10 for the anxiety subscale^11^.

### Tinetti Falls Efficacy Scale

The Tinetti Falls Efficacy Scale (TFES), developed by Tinetti et al.^12^, was translated into Turkish by Erdem and Emel^14^. This scale consists of 10 items, each assessing the level of confidence in performing daily activities. Each item is scored between 1 (very confident) and 10 (not confident at all). The total score is the sum of these item scores, with higher scores indicating a greater fear of falling.

### Sample size calculation

Sample size calculation, based on Gunendi et al.’s^3^ study, used TSK scores with means of 39±6.3 for healthy individuals and 43±8.6 for osteoporosis patients, resulting in an effect size of 0.53. With an alpha of 0.05 and a power of 0.80, 56 participants per group were needed. Our study included 60 participants per group, with calculations done using the G*Power software version 3.1.9.4.

### Statistical analysis

Data normality was assessed with the Kolmogorov-Smirnov test. Quantitative data are presented as mean and standard deviation, while qualitative data are presented as frequencies and percentages. For normally distributed data, independent sample t-tests will be used; otherwise, Mann-Whitney U tests will be used. The chi-square test will assess qualitative data. A p<0.05 is considered significant. Pearson and Spearman correlation tests will be used for normally and non-normally distributed data, respectively.

## RESULTS

Table 1 presents the study’s descriptive data. The mean age was 61.08±7.20 years in the osteoporosis group and 58.72±6.96 years in the control group, with no significant age difference (p>0.05). BMI was higher in the control group. Both groups were similar in education, occupation, marital status, smoking habits, and pain characteristics. Hypothyroidism and diabetes were more common in the control group, but there were no significant differences in hypertension and coronary artery disease (Table 1).

**Table 1:** Descriptive data.

		Osteoporosis			Control			p-value
		Mean	±SD/ n%	Median (min–max)	Mean	±SD/ n%	Median (min–max)	
Age		61.08	±7.20	61 (45–79)	58.72	±6.96	57 (50–76)	0.053^m^
BMI		26.19	±3.84	25.52 (17.9–35.7)	30.80	±5.18	30.45 (18.73–41.62)	***0.000*** ^t^
Education duration		7.87	±3.93	5 (0–16)	7.88	±3.57	5 (0–16)	0.863^m^
Occupation	Employed	1	1.67%		3	5.00%		0.595^x^
	Retired	22	36.67%		21	35.00%		
	Housewife	37	61.67%		36	60.00%		
Marital status	Married	45	75.00%		46	76.67%		0.831^x^
	Single	15	25.00%		14	23.33%		
Hypertension	None	39	65.00%		36	60.00%		0.572^x^
	Present	21	35.00%		24	40.00%		
Diabetes	None	54	90.00%		45	75.00%		***0.031*** ^x^
	Present	6	10.00%		15	25.00%		
CAD	None	52	86.67%		56	93.33%		0.224^x^
	Present	8	13.33%		4	6.67%		
Hypothyroidism	None	54	90.00%		42	70.00%		***0.006*** ^x^
	Present	6	10.00%		18	30.00%		
Smoking	None	46	76.67%		49	81.67%		0.500^x^
	Present	14	23.33%		11	18.33%		
Exercise	None	49	81.67%		51	85.00%		0.624^x^
	Present	11	18.33%		9	15.00%		
Femur BMD		0.73	±0.09	0.73 (0.53–0.99)	0.92	±0.11	0.89 (0.80–1.46)	***0.000*** ^m^

^m^Mann-Whitney U test

^t^independent two-sample t-test

^x^chi-square test. SD: standard deviation, BMI: body mass index, CAD: coronary artery disease, GIS: gastrointestinal system, BMD: bone mineral density. Statistically significant values are indicated in bold.

In the osteoporosis group, TSK scores were significantly higher compared to the control group (p<0.05). However, no significant differences were found between the two groups in terms of QUALEFFO-41 pain, physical function, general health, mental health, and total scores (p>0.05). HADS depression and anxiety scores, as well as TFES scores, were similar between the two groups (Table 2).

**Table 2:** Quality of Life Questionnaire of the European Foundation for Osteoporosis, Tampa Scale for Kinesiophobia, Hospital Anxiety and Depression Scale, and Falls Efficacy Scale scores.

	Osteoporosis		Control		p-value
	Mean±SD	Median (min–max)	Mean±SD	Median (min–max)	
QUALEFFO-41 pain score	43.25±23.81	45 (0–90)	45.09±27.07	45 (0–100)	0.693^t^
QUALEFFO-41 physical function score	35.08±15.45	33.86 (7.60–65.52)	37.37±17.54	36.90 (4.17–77.08)	0.450^t^
QUALEFFO-41 general health score	50.30±13.32	50 (16.66–75)	54.03±16.22	50 (16.66–100)	0.408^m^
QUALEFFO-41 mental function score	45.97±14.27	48.61 (11.11–72.22)	44.63±14.36	44.44 (8.33–77.77)	0.609^t^
QUALEFFO-41 total score	40.49±13.43	41.33 (16.64–70.51)	41.36±14.61	42.53 (9.15–67.86)	0.734^t^
TSK score	41.73±5.48	41 (26–54)	38.92±5.94	40 (23–52)	***0.018*** ^m^
HADS depression score	7.10±3.47	7 (1–15)	6.07±3.74	6 (0–14)	0.128^m^
HADS anxiety score	8.43±3.58	8 (3–19)	8.27±4.71	8 (0–19)	0.632^m^
Tinetti Falls Efficacy Scale	26.47±16.86	20.50 (10–85)	27.40±16.85	20 (10–81)	0.596^m^

^t^Independent two-sample t-test;

^m^Mann-Whitney U test. QUALEFFO-41: Quality of Life Questionnaire of the European Foundation for Osteoporosis, TSK: Tampa Scale for Kinesiophobia, HADS: Hospital Anxiety and Depression Scale, SD: standard deviation. Statistically significant values are indicated in bold.

There were no significant relationships between the TSK scores and age, BMI, femoral neck bone mineral density, lumbar L1-L4 and femoral neck T-scores, or QUALEFFO-41 pain, general health, mental health, and total scores. However, the level of kinesiophobia was positively correlated with TFES scores (r=0.278, p=0.002) and QUALEFFO-41 physical function scores (r=0.185, p=0.043). No significant relationship was found between TSK scores and HADS anxiety and depression scores (Table 3).

**Table 3:** Correlation between Tampa Scale for Kinesiophobia and other parameters.

	TSK	
	Rho	p-value
Age	0.156	0.089
BMI	-0.044	0.635
Femoral bone mineral density	-0.141	0.123
Lumbar DXA score	-0.176	0.055
Femoral DXA score	-0.160	0.080
QUALEFFO-41 pain score	0.141	0.125
QUALEFFO-41 physical function score	***0.185*** ^*^	***0.043***
QUALEFFO-41 general health score	0.065	0.478
QUALEFFO-41 mental function score	0.084	0.362
QUALEFFO-41 total score	0.170	0.063
HADS depression score	0.018	0.842
HADS anxiety score	0.104	0.257
Tinetti Falls Efficacy Scale	***0.278*** ^**^	***0.002***

Spearman correlation test. BMI: body mass index, DXA: dual-energy X-ray absorptiometry, QUALEFFO-41: Quality of Life Questionnaire of the European Foundation for Osteoporosis, TSK: Tampa Scale for Kinesiophobia, HADS: Hospital Anxiety and Depression Scale. Statistically significant values are indicated in bold.

*Correlation is significant at the 0.05 level (two-tailed).

**Correlation is significant at the 0.01 level (two-tailed).

## DISCUSSION

In this study, the level of kinesiophobia was found to be significantly higher in patients with osteoporosis compared to the control group. It was also observed that patients with higher levels of kinesiophobia had a greater fear of falling. Additionally, a positive correlation was found between kinesiophobia scores and QUALEFFO-41 physical function subscale scores.

Mısırcı et al.^15^ found similar kinesiophobia levels in osteoporotic and osteopenic groups, both higher than the control group, and reported positive correlations with quality of life, fear of falling, depression, and anxiety. Similarly, another study^3^ observed higher kinesiophobia in osteoporotic patients versus controls. Our study supports these findings, showing higher kinesiophobia in the osteoporosis group. Excluding participants with painful musculoskeletal disorders suggests that kinesiophobia can arise independently of pain, aligning with the literature that links it directly to osteoporosis^3,15^.

In the literature, studies have found correlations between kinesiophobia levels and age and BMI in knee osteoarthritis and chronic low back pain^16,17^. Turgay et al.^18^ reported no significant relationship between kinesiophobia and BMI in postmenopausal osteoporotic patients but did find a positive correlation between age and kinesiophobia. Mısırcı et al.^15^ found correlations between kinesiophobia and age and BMI only in the osteopenic group. However, our study found no association between kinesiophobia and age or BMI.

In our study, fear of falling was similar between the osteoporosis and control groups, although kinesiophobia levels were positively correlated with fear of falling. Contrary to our results, other studies found higher fear of falling in the osteoporosis group^19,20^. Mısırcı et al.^15^ reported significantly higher fear of falling in both osteoporotic and osteopenic groups compared to controls, with no significant difference between the two groups. They also found a positive correlation between kinesiophobia and fear of falling. Similarly, a study on rheumatoid arthritis patients showed a significant relationship between kinesiophobia and fear of falling, aligning with our results^21^.

In our study, the osteoporosis and control groups were found to be similar in terms of depression and anxiety symptoms. Similarly, the study by Gunendi et al.^3^ reported no significant differences in depression and anxiety symptoms between the osteoporosis and control groups, consistent with our findings. However, Kashfi et al.^22^ found significantly higher depression scores in the osteoporotic group compared to the control group. Additionally, a study involving 11,603 adults reported that individuals with osteoporosis exhibited 1.73 times more depressive symptoms compared to those without osteoporosis^23^. Other studies in the literature have also reported higher levels of depression and anxiety symptoms in patients with osteoporosis^15^.

Our study found no significant relationship between kinesiophobia and depression or anxiety scores. This contrasts with Mısırcı et al.^15^, who reported higher depression and anxiety in patients with high kinesiophobia. Similar correlations have been observed in conditions like ischemic stroke^24^ and systemic lupus erythematosus^7^. A study of elderly nursing home residents also found a positive correlation between kinesiophobia and anxiety and depression symptoms^25^. However, a study on fibromyalgia patients did not find an association between kinesiophobia and depression^4^.

In Altuğ et al.’s study^5^, kinesiophobia scores were negatively correlated with physical function subscale scores of quality of life in chronic low back pain, similar to our findings. However, we did not find significant relationships with pain, general health, or mental health parameters. Gunendi et al.^3^ reported that higher kinesiophobia levels were associated with a decline in quality of life in osteoporosis patients. Conversely, another study^15^ found a positive correlation between kinesiophobia and total QUALEFFO-41 scores, without analyzing subscales separately. Turgay et al.^18^ observed a positive correlation between kinesiophobia and both total QUALEFFO-41 scores and all subscales.

One of the limitations of our study is its single-center cross-sectional design. Although the sample size was calculated at the beginning of the study, there is a need for studies with larger sample sizes. Additionally, the use of the TSK, which is not specific to osteoporosis, also constitutes a limitation. However, a strong aspect of our study is the relatively small number of studies comparing kinesiophobia in osteoporosis patients with depression, anxiety, fear of falling, and quality of life in a control group.

In conclusion, our research highlights that kinesiophobia levels are higher in the osteoporotic patient group compared to healthy controls. As restricted physical activity can lead to decreased bone mineral density and an increased risk of fractures, early identification of kinesiophobia and implementing measures to ensure patients remain active is crucial for the treatment and rehabilitation process.
